# 3D-Printed Potentiometric Multicells for Enhanced Analytical Performance of Solid Contact Ion-Selective Electrodes

**DOI:** 10.1021/acs.analchem.5c06253

**Published:** 2025-12-11

**Authors:** Dario Torricelli, Daniel Rojas, María Cuartero, Gastón A. Crespo

**Affiliations:** † UCAM-SENS, 16728 Universidad Católica San Antonio de Murcia, UCAM HiTech, Avda. Andres Hernandez Ros 1, 30107 Murcia, Spain; ‡ Department of Chemistry, 7655 KTH Royal Institute of Technology, Teknikringen 30, SE-114 28 Stockholm, Sweden; § The Institute of Biotechnology and Genetic Engineering, Chulalongkorn University, Bangkok 10330, Thailand

## Abstract

The sensitivity of ion-selective electrodes (ISEs) that are operated by equilibrium potentiometry is determined by the well-known Nernst equation. An elegant concept that involved connecting various potentiometric cells in series was demonstrated in early reports with the aim of increasing the sensitivity of ISEs by multiplying the theoretical slope by the number of cells used. In the 1980s and 1990s, the concept of cells connected in series (CCS) was demonstrated to be capable of amplifying the potentiometric signal, enhancing the determination of selected analytes. Nevertheless, the use of bulky electrodes and cumbersome methods for sensor fabrication restricted its applicability to modern analytical contexts. Hereby, we revive and modernize this overlooked concept by introducing 3D-printed potentiometric multicells (3DP-PMCs) that integrate solid-contact ISEs and solid-state reference electrodes into compact, interconnected architectures. Owing to the design flexibility and reproducibility of 3D printing, the 3DP-PMC platform enables slope multiplication by up to 8-fold, achieving sensitivities of 475 ± 12 mV dec^–1^. Importantly, this enhanced performance is maintained across narrow concentration intervals, with the octuple-cell configuration allowing detection of 0.1 mM concentration changes (equivalent to a 2% change) not achievable with an individual cell configuration. This work demonstrates, for the first time, the practical translation of the CCS principle into a miniaturized, modular, and easily manufacturable format, paving the way for its integration into microfluidic, wearable, and point-of-care devices.

## Graphical abstract

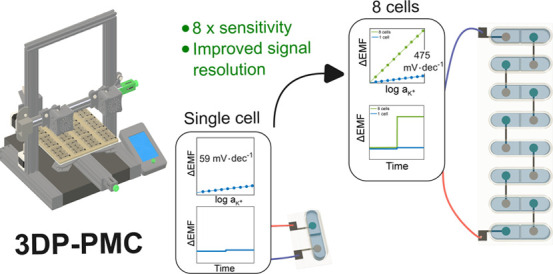

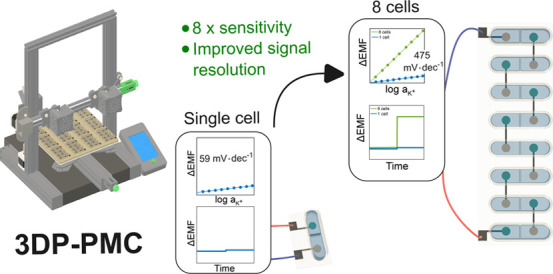

Ion-selective electrodes (ISEs) have traditionally relied on internal solution (IS) electrodes. While effective, these designs are bulky, fragile, and almost unsuitable for modern analytical devices. The introduction of solid-contact ISEs (SC-ISEs) eliminates the internal solution and uses a solid ion-to-electron transducing layer, allowing for miniaturized, robust, and scalable sensors.^1,2^ This innovation made SC-ISEs a key architecture for today’s applications relying on ion detection, where size, stability, and integration into electronics are musts.

Despite this progress, a fundamental constraint of ISEs functioning under zero current settings (i.e., potentiometric mode, equilibrium conditions) is their fixed sensitivity, as dictated by the Nernst equation. The voltage measured in an ion-selective electrode is directly proportional to the logarithm of the ion activity, with a slope of 59.2/z mV per decade at 25 °C (z = ion charge). An improvement in sensor sensitivity is anticipated to reduce experimental errors, particularly when distinguishing and quantifying very narrow concentration changes of the analyte. This is especially pertinent for environmental, wearable, and point-of-care applications, because ion concentrations often lie within a small level range (and in the millimolar scale).^3^

Diverse interrogation techniques have been proposed to enhance the sensitivity of ion-selective electrodes under nonequilibrium settings.^4^ In this regard, ion-transfer voltammetry has been extensively developed in recent years. This approach relies on the oxidation–reduction of an electroactive transducer layer (e.g., a conducting polymer) in connection to the traditional ion-selective membrane used in potentiometric ISEs. This generates a charge imbalance that leads to ion transfer at the membrane/sample interface. Two response regimes are typically observed based on the concentration range of the ion analyte in the sample solution.^5,6^ At low sample concentrations (nM−μM), the peak current exhibits a linear dependence on concentration; however, at high concentrations (μM–mM), the peak potential follows a Nernstian shift with the logarithmic ion analyte activity. A primary feature of this technology is the capability to detect many ions with a single sensor utilizing a membrane containing various ionophores.^7^ Alternatively, notable progress has been achieved using a coulometric readout of ISEs. The application of a certain potential differences produces current spikes/decays that can be converted into charge, revealing a linear relationship with the ion activity that exceed the Nernstian slope of potentiometric ISEs.^5^ Due to this improved sensitivity, Bakker’s group reported pH changes in mpH units, while Mikhelson’s group was capable of detecting changes in Ca^2+^ concentration below 1%.^8,9^ However, the response time of the sensor is dependent upon electrode capacitance, membrane resistance, and the magnitude of the concentration change.^10,11^ Furthermore, such nonequilibrium approaches generally require complex instrumentation, exhibit response times dependent on electrode capacitance and membrane resistance, and are less suited to real-time monitoring compared to classical equilibrium potentiometry.

Alternatively, some strategies to amplify potentiometric signals under equilibrium conditions have also been investigated. In 1983, Stepak^12^ first described the cell connected in series (CCS) concept. In this configuration, series interconnection is created by connecting the indicator electrode of a cell to the reference electrode of the next cell, with the overall connection to the potentiometer done with the first indicator and last reference electrode. Using this approach, the voltage measured in the potentiometer was revealed to be the sum of the potential provided by each of the cells. Stepak demonstrated the concept with a potentiometric titration of Cl^–^.^12^ Later, the concept was translated to IS electrodes containing plasticized K^+^-selective PVC membranes by Suzuki in 1987.^13^ In 1989, Cheng presented a patent describing the same principle.^14^ During the early 1990s, further adaptations appeared: Wanli presented in 1991 a five-cell probe for quinine detection, where sample solution was held in separate compartments by wax-defined hydrophobicity,^15^ while Hibbert and co-workers applied the multicell principle to segmented flow analysis.^16^ After these early demonstrations, the concept remained largely dormant for decades. Only recently, in the 2020s, Zdrachek and co-workers revived the CCS approach, applying it to both monovalent (K^+^, NO_3_^–^) and divalent (Ca^2+^, CO_3_^2–^) ions using macro-sized SC-ISEs.^17^ Interestingly, it was demonstrated that the approach can be applied not only to monovalent ions but also to divalent ones. These studies highlighted the universality of the concept but relied on large beaker-based setups with glassy carbon electrodes, formats not easily translating to modern sensing technologies such as wearable, portable, or point-of-care systems.

Recently, our group reported the use of 3D printing for reproducible fabrication of SC-ISEs.^18^ Building on this foundation, we now revisit the CCS principle through additive manufacturing. The design flexibility, modularity, and efficiency of 3D printing enable the realization of interconnected SC-ISE arrays in a compact and robust format. In this work, we present the first 3D-printed potentiometric multicell (3DP-PMC) with the following unique features: (i) slope amplification by up to 8-fold, (ii) high-resolution detection of potassium ions within physiologically relevant ranges, and (iii) the potential for future integration into microfluidic and wearable platforms.

## Results and Discussion

Herein, we describe a 3DP-PMC comprising 2 to 8 CCS, each containing indicator and reference electrodes. The device is fabricated by multimaterial FFF 3D printing, and the sensing and reference membranes are further added manually with minimal manipulation (Figure 1a). To better show the structure of the 3DP-PMC, an exploded view of the double configuration is shown in Figure 1b. By extending the serial connection as shown in Figure 1c, the quadruple, sextuple, and octuple configurations can be fabricated. The solid-contact ISEs were prepared following our previously reported protocol for 3DP-SC-ISEs,^18^ while the reference electrodes were obtained by manual deposition of an Ag/AgCl layer and a PVB-based reference membrane. Full fabrication protocols are provided in the Supporting Information. By extension of the printed interconnections, the minimal double-cell design (Figure 1b) can be expanded to quadruple, sextuple, and octuple configurations. The expected outcome from this configuration is an enhanced sensitivity with a linear increase in the potentiometric slope, as described in eq 1. Figure 1d shows the expected ΔEMF for the different cell configurations, providing an enhanced sensitivity. This improved signal allows for better resolution in detecting small concentration changes, if the noise does not increase proportionally with the signal. Consequently, the signal-to-noise (S/N) ratio improves, enabling precise concentration discrimination in multicell configurations that would not have been possible with a single cell (Figure 1e).

1 $$EMF=n_{cells}\cdot E^{0}+n_{cells}\cdot \frac{RT}{zF}\cdot \log a_{ion}$$

**Figure 1:**
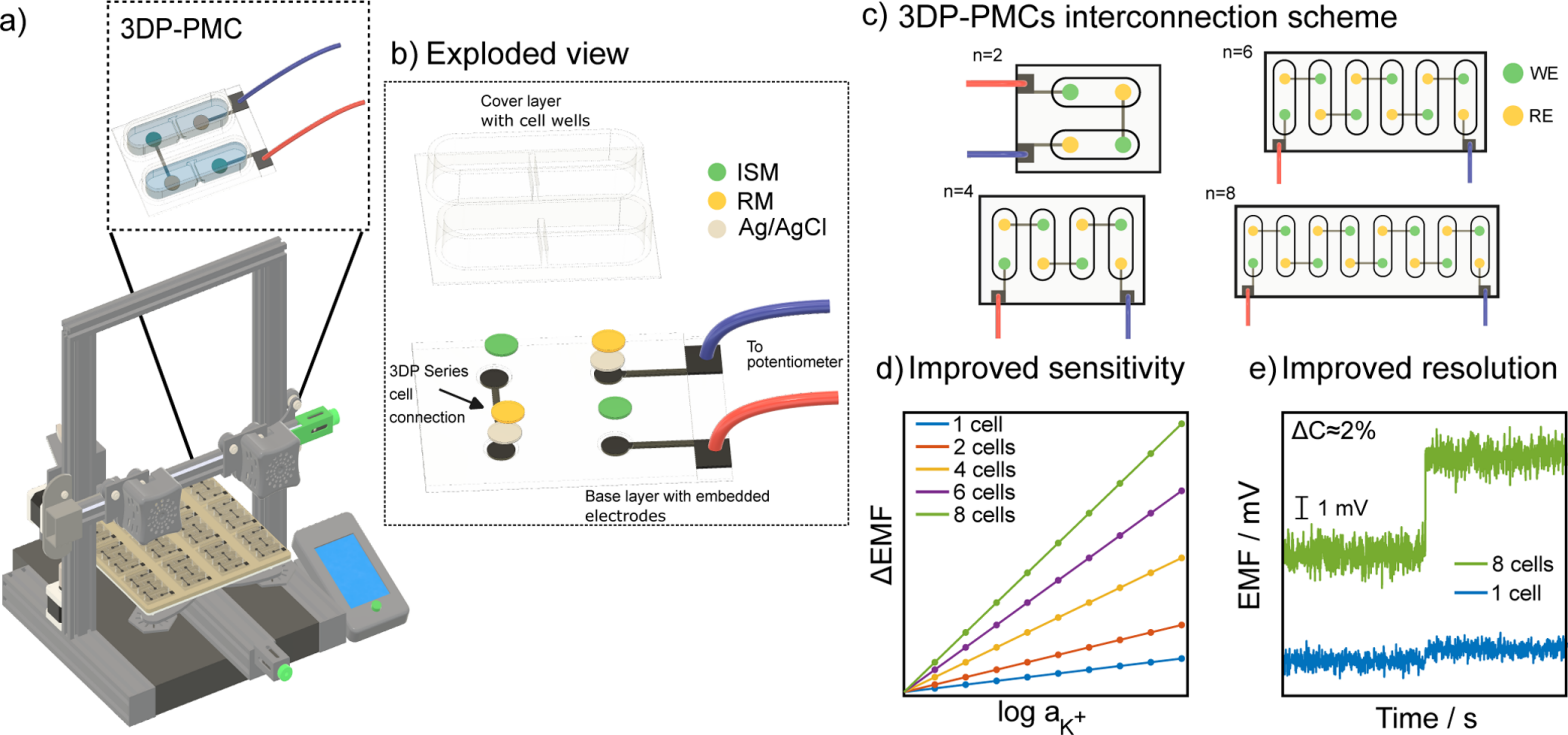
Schematic representation of the 3DP-PMC concept. (a) Development of the sensors using multimaterial fused filament fabrication (FFF). (b) Exploded view of the 3DP-PMC showing the different components. (c) Scheme of the series connection in double, quadruple, sextuple, and octuple configurations. (d) Simulated signals illustrating the expected increase in sensitivity by serial connection of cells. (e) Enhanced resolution for small concentration changes owing to the large improvement of the signal-to-noise ratio (S/N) as a function of the increasing number of cells.

The electrochemical response of the different 3DP-PMC configurations was first evaluated by using K^+^ solutions spanning from 10^–5^ to 10^–1^ M. Figure S1 displays the time traces and average calibration curves for three different devices of each configuration (individual, double, quadruple, sextuple, and octuple). All configurations showed linear trends with slopes close to the Nernstian value. The specific calibration parameters are summarized in Table 1. For the sake of clarity, Figure 2a compiles the average calibration plots obtained with three equally prepared devices per configuration. As expected, both the EMF and the slope increased proportionally with the number of interconnected cells. In particular, the linear dependence between the slope and cell number (Figure 2b) displayed a slope of 59.4 ± 0.5 mV·(dec·cell)^−1^, confirming the preservation of Nernstian behavior in all cases. Similarly, the standard potential (E^0^) scaled linearly with the number of cells (Figure 2c), with a slope of 688 ± 1 mV·cell^–1^, closely matching the E^0^ value of a single cell (Table 1). These results demonstrate the experimental validity of eq 1.

**Figure 2:**
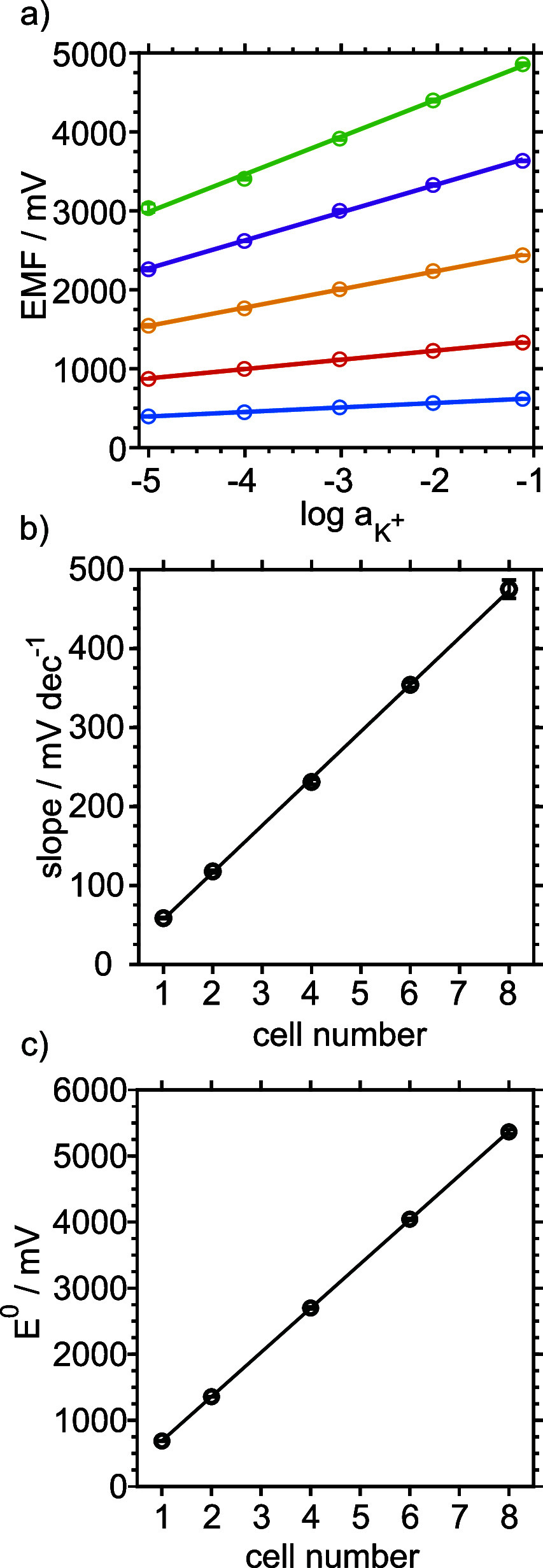
(a) Potentiometric response of individual and the 3DP-PMCs as a function of the logarithm of potassium ion activity. Single (blue), double (orange), quadruple (yellow), sextuple (purple), and octuple (green) cells are presented. (b) and (c) show the slope (mV·dec^–1^) and standard potential (E^0^, mV), respectively, as a function of the cell number. Measurements presented correspond to *n* = 3 different devices for each of the single and multicell configurations.

**Table 1:** Calibration Parameters Observed for Individual and 3DP-PMCs in the Range of Potassium Ion Activity from 10^–5^ and 10^–1.11^ ^a^

Cell number	Slope (mV·dec^–1^)	*E* ^0^ (mV)
1	57.4 ± 0.3	683 ± 5
2	118 ± 2	1357 ± 3
4	231 ± 3	2700 ± 15
6	354 ± 3	4043 ± 16
8	475 ± 12	5365 ± 32

a Measurements presented correspond to n = 3 different devices for each of the single and multicell configurations.

The analytical advantage of slope multiplication becomes evident when resolving small concentration differences. To illustrate this, K^+^ levels within the physiologically relevant ranges (2–10 mM in sweat; 3.5–5 mM in blood)^19,20^ were measured using concentration steps of 0.1, 0.25, 0.5, 1, and 5 mM. Figure 3 shows the calibration responses of single-, quadruple-, and octuple-cell devices. While all configurations retained linearity, the ability to resolve small changes improved substantially with higher cell numbers. For example, a 0.1 mM step (2% concentration change) was barely distinguishable from noise in the single-cell configuration but produced a clear EMF increase in the quadruple and octuple configurations. This is clearly visible in the time traces shown in the left panel of Figure 3 (0.1 mM step between 4.5 and 4.6 mM), whereas the single-cell signal is indistinguishable from baseline noise and the multicell devices provided well-defined responses. At 0.25 mM (6% change), the single cell provided marginally detectable signals, while quadruple and octuple devices achieved clear detection compared to the noise level.

**Figure 3:**
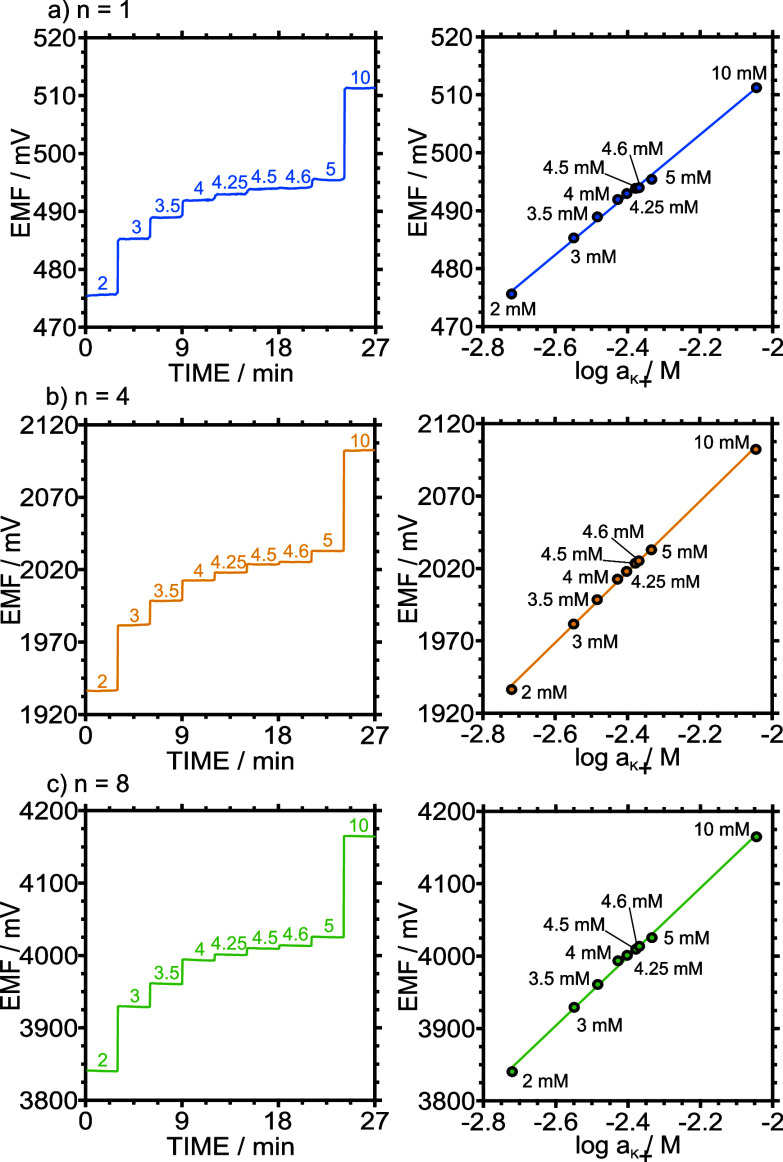
Potentiometric response for a narrow range of potassium ion concentration covering the physiological range in biofluids. The configurations include (a) a single, (b) quadruple, and (c) octuple 3DP-PMC setups. Left side shows the time traces containing the corresponding concentration expressed in mM for each of the steps, whereas the right side shows the corresponding linear regression using the signal for each of the concentrations.

To systematically quantify the resolution improvement in measuring different concentration solutions, signal-to-noise (S/N) ratios were calculated. For the signal (S), we used the EMF difference between the two tested concentrations with the standard deviation of the baseline EMF response as an estimation of the noise (N). Figure S2 first compares the noise levels for the different multicell configurations, demonstrating a clear increase of noise levels with the number of cells. Nevertheless, despite the rise in the noise levels, Figure S3 illustrates that concentration differentiation over the noise improves with increasing cell number as the amplified signal escalates more significantly than the noise.

The experimental S/N increases with the number of cells connected in series, following the expected $\sqrt{n_{cells}}$ prediction for uncorrelated noise. In this situation, the noise contributions of individual isolated cells can be considered statistically independent, so that while the total signal increases proportionally to the number of cells, the overall noise increases as $\sqrt{n_{cells}}$ , leading to the nonlinear S/N improvement reported. This relationship is derived from the statistical theory for independent noise sources resulting in that the standard deviation (noise amplitude) increases as $\sqrt{n_{cells}}$ .^21,22^ Figure S4 illustrates the linear relationship between the S/N and $\sqrt{n_{cells}}$ observed for all concentrations, and Table 2 summarizes the results obtained. Note that in all cases the experimental S/N exceeds the theoretical prediction, particularly at low ΔC (0.1–0.25 mM), suggesting that correlated behavior or partial common-mode noise cancellation enhances precision beyond the purely statistical limit. At higher ΔC (0.5–1 mM), the S/N trend approaches the ideal $\sqrt{n_{cells}}$ trend, consistent with random noise dominance. These results confirm that signal amplification and partial noise averaging contribute to improving the S/N outcomes.

**Table 2:** S/N Calculated for Different Concentration Differences (ΔC) Expressed in mM as a Function of the Number of Cells^a^

ΔC (mM)	***n***_cells_	$\sqrt{n_{\mathrm{cells}}}$	Experimental S/N	Expected S/N
0.1	1	1.00	1	1
	4	2.00	5	2
	8	2.83	7	3
0.25	1	1.00	4	4
	4	2.00	18	8
	8	2.83	15	11
0.5	1	1.00	14	14
	4	2.00	45	28
	8	2.83	60	40
1	1	1.00	39	40
	4	2.00	118	78
	8	2.83	169	110

a Expected S/N is calculated using the $\sqrt{n_{cells}}$ scaling considering the S/N for n = 1 cells.

While all configurations were adequate for larger steps (≥0.5 mM), multicell devices provided markedly higher S/N, particularly at the lowest increments. For 0.1 mM, the single-cell device yielded S/N = 1 (not detectable), whereas quadruple and octuple configurations reached values >3 (detectable). At 0.25 mM, S/N rose from 4 (single) to 15–18 (multicells), enabling reliable quantification. This demonstrates the unique advantage of 3DP-PMCs for high-resolution potentiometric sensing.

Finally, these findings confirm that slope multiplication in 3DP-PMCs not only preserves the Nernstian response but also translates into practical analytical benefits: enhanced resolution, improved S/N, and reliable detection of concentration differences obscured in conventional single-cell devices.

## Conclusions

We presented an approach consisting of connecting several ion-selective cells in series within a compact, 3D-printed platform (3DP-PMC). Utilizing the flexibility of additive manufacturing, we engineered and produced multicell devices with up to eight interconnected cells, each consisting of a solid-contact ion-selective and solid-state reference electrode. The overall EMF of the system increased linearly with the number of cells, influencing both the slope and the intercept (E^0^). Consequently, we achieved a predictable enhancement in the analytical sensitivity. Moreover, the signal-to-noise ratio is improved in the multicell configuration, leading to greater resolution for detecting small variations in ion concentration—particularly within physiologically relevant ranges. Notably, the multicell configuration clearly resolved concentration steps that conventional single-cell devices could not distinguish. The simplicity, reproducibility, and customizability features of the 3DP-PMCs provide it as a compelling candidate for the next generation of potentiometric sensors, particularly in high-resolution applications such as wearable and point-of-care diagnostics. Future developments will concentrate on miniaturizing the concept and incorporating it into autonomous and multiplexed sensing systems while also enhancing the signal-to-noise ratio through larger multicell arrangements.

## Supplementary Material



## Notes

Experimental details, schematics of the 3DP-PMC, calibrations of the different configurations of 3DP-PMC, and time-traces of the 3DP-PMC in the narrow concentration range (PDF)
