# Supplementary material for: 3D-Printed Potentiometric Multicells for Enhanced Analytical Performance of Solid Contact Ion-Selective Electrodes
Source: Anal Chem. 2025 Dec 11;97(50):27532–6. doi: 10.1021/acs.analchem.5c06253 (PMC12750411; doi:10.1021/acs.analchem.5c06253)
Supplement: Supplementary file 1 [file ac5c06253_si_001.pdf]

Supporting Information

3D-Printed Potentiometric Multi-cells for Enhanced Analytical Performance of Solid Contact Ion-Selective Electrodes.

Dario Torricelli[1⊥], Daniel Rojas[1⊥], María Cuartero[1,2], Gastón Crespo[1,2,3,*]

[1]UCAM-SENS, Universidad Católica San Antonio de Murcia, UCAM HiTech, Avda. Andres Hernandez Ros 1, 30107, Murcia, Spain
[2]Department of Chemistry, KTH Royal Institute of Technology, Teknikringen 30, SE-114 28, Stockholm, Sweden.
[3]The Institute of Biotechnology and Genetic Engineering, Chulalongkorn University, Bangkok, 10330, Thailand.

*Corresponding authors: gacp@kth.se

Table of Contents

Experimental Section

Reagents and Materials. SI-3

Design and Fabrication of 3D-Printed Multi-Cell Devices (3DP-PMC). SI-3

Electrode Modification. SI-3

Potentiometric Measurements. SI-3

Figures

Figure S1. .... SI-4

Figure S2. .... SI-5

Figure S3. .... SI-5

Figure S4. .... SI-6

References SI-7

Experimental Section

Reagents and Materials.

Polyvinyl chloride (PVC), Bis(2-ethylhexyl) sebacate (DOS), sodium tetrakis[3,5-bis(trifluoromethyl)phenyl] borate (NaTFPB), Tetrahydrofuran (THF) were of Selectophore grade (Sigma-Aldrich). Polyvinyl butyral (PVB) and Ag/AgCl (60/40) paste for screen printing were also purchased from Sigma-Aldrich. Methanol (>98.5%) and Sodium chloride (99.5-100.5%, AnalaR NORMAPUR®) were purchased from VWR. The filaments used for 3D printing of the devices are Polylactic acid filament (PLA, Smartfil, Smart Materials 3D) as insulator material and Carbon black filled polylactic acid filament (CB-PLA) (Protopasta CDP11705, Protoplant) as electrically conductive material.

Design and Fabrication of 3D-Printed Multi-Cell Devices (3DP-PMC).

The 3DP-PMC devices were designed in Fusion 360 (Autodesk, USA), exported as STL files, and processed with PrusaSlicer (Prusa Research, Czech Republic). Printing was carried out on a multi-tool Prusa XL 3D printer equipped with two independent toolheads, each fitted with a 0.4 mm brass nozzle, for deposition of PLA and CB-PLA. Printing parameters were optimized for device reproducibility, employing 100% infill, an extrusion multiplier of 1.1, and a print speed of 25 $mm \cdot s^{-1}$. Nozzle temperatures were set to 230 °C (PLA) and 240 °C (CB-PLA), with bed temperatures of 60 °C and 90 °C, respectively. The printed structures consisted of a 0.8 mm-thick base layer containing 0.4 mm-deep grooves to host conductive tracks and electrodes (0.4 mm thickness, Figure 1a). The specific pattern of grooves and conductive pathways was adapted to the number of interconnected cells. A PLA cover layer was subsequently printed as an insulating element, incorporating cavities to host the ion-selective membranes and Ag/AgCl reference layers, as previously described.[1] In addition, a PLA well was integrated above the electrode assembly to confine the conditioning and measurement solutions. Although described here as separate layers for clarity, the device is fabricated monolithically in a single print step, ensuring seamless integration of insulating and conductive components. Depending on the intended configuration, designs were extended from the basic double-cell unit to obtain 4-, 6-, or 8-cell 3DP-PMCs (Figure 1c).

Electrode Modification.

Solid-State Reference electrodes (3DP-SS-REs):

CB-PLA electrodes were first coated with Ag/AgCl paste and cured at 60 °C for 30 min. A reference membrane (RM) solution was prepared by dissolving 78 mg of PVB and 50 mg of NaCl in 1 mL methanol. Electrodes were coated with five sequential 10 µL layers of RM solution, allowing ≥20 min for evaporation between layers, as reported previously.[1]

Solid-Contact Indicator electrodes (3DP-SC-ISEs):

A potassium-selective cocktail was prepared containing 1 wt% potassium ionophore I, 0.5 wt% NaTFPB, 65 wt% DOS, and 32.5 wt% PVC. A total of 100 mg of this mixture was dissolved in 1 mL THF. Each electrode was coated with 50 µL of the cocktail to form the ion-selective membrane, following previous procedures.[2]

Potentiometric Measurements.

Measurements were carried out using a 16-channel high-input-impedance potentiometer (10^{15} Ω, EMF16, Lawson Laboratories, Inc.). Prior to perform any potentiometric measurement, an overnight conditioning step was performed by filling each half of the 3DP-PMC with 250 µL of the corresponding conditioning solution (3M KCl for 3DP-SS-RE and 10 mM KCl for 3DP-SC-ISE. Calibration curves for K^+ were obtained by sequentially exchanging standard KCl solutions in the measurement well, rinsing with Milli-Q water, and drying under N_2 between measurements. For small concentration increments, aliquots of standard KCl solution were added directly to 1.5 mL of sample solution, followed by homogenization with a pipette tip.

Figures

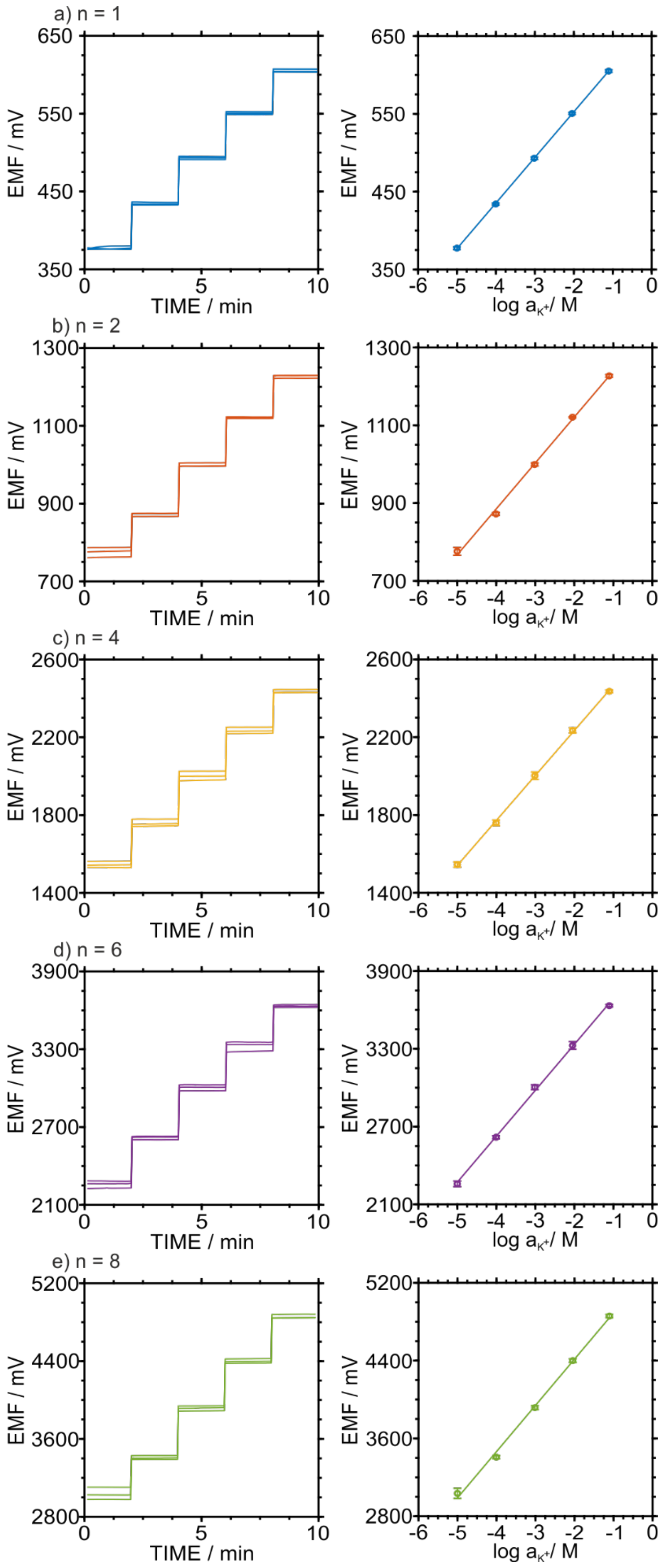

Figure S1. Time trace and calibration plot of three identically prepared 3DP-PMC: (a) individual cell (b) double cell (c) quadruple cell (d) sextuple cell and (e) octuple cell.

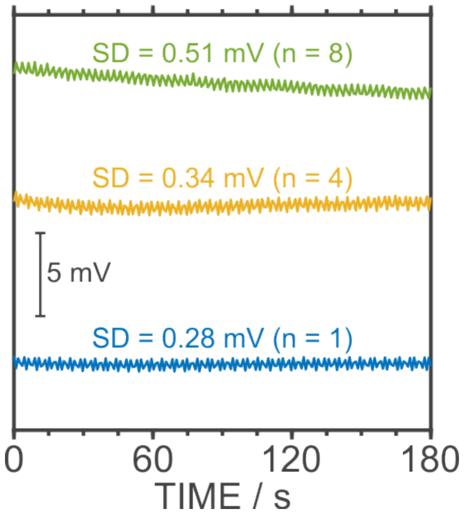

Figure S2. Noise levels of a concentration of 3.5 mM of K^+ in single, quadruple and octuple cell configurations.

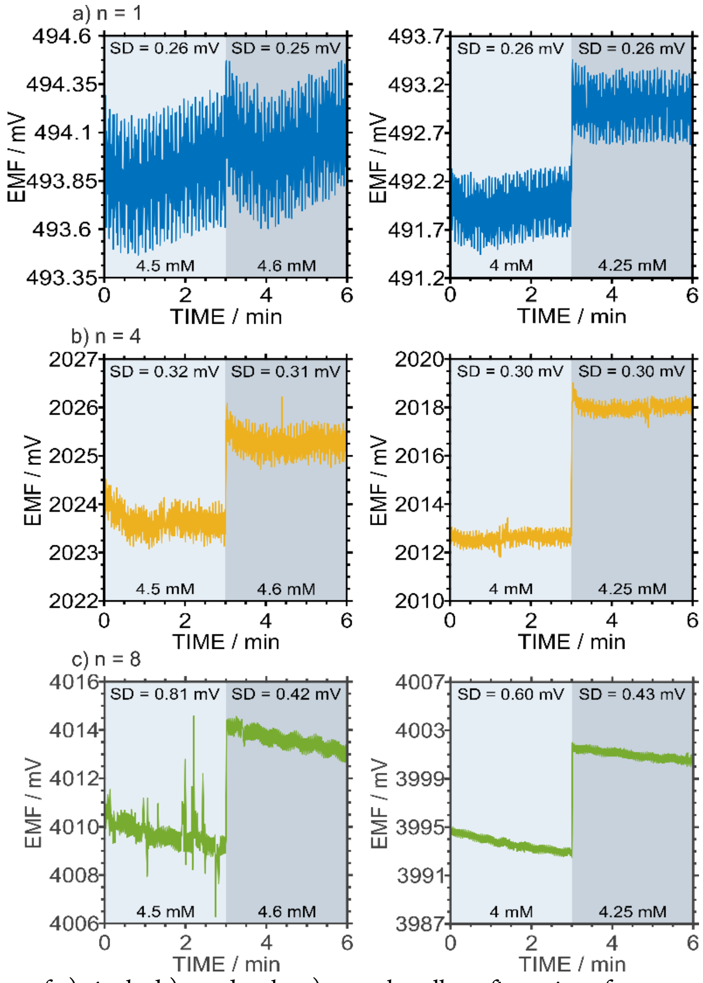

Figure S3. Time traces of a) single, b) quadruple, c) octuple cell configurations for a concentration jump of 0.1 mM (left panel) and 0.25 mM (right panel).

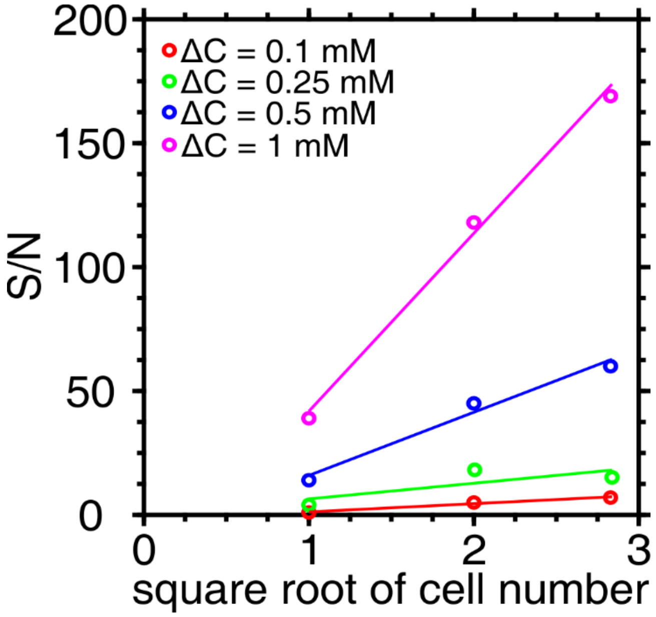

Figure S4. Dependence on the S/N with the $\sqrt{n_{cells}}$ for the different concentration steps 0.1, 0.25, 0.5 and 1 mM

References

(1) Guinovart, T.; Crespo, G. A.; Rius, F. X.; Andrade, F. J. A Reference Electrode Based on Polyvinyl Butyral (PVB) Polymer for Decentralized Chemical Measurements. *Anal Chim Acta* 2014, *821*, 72–80..

(2) Rojas, D.; Torricelli, D.; Cuartero, M.; Crespo, G. A. 3D-Printed Transducers for Solid Contact Potentiometric Ion Sensors: Improving Reproducibility by Fabrication Automation. *Anal Chem* 2024.
